# Spatial organization of the chicken beta-globin gene domain in erythroid cells of embryonic and adult lineages

**DOI:** 10.1186/1756-8935-5-16

**Published:** 2012-09-07

**Authors:** Sergey V Ulianov, Alexey A Gavrilov, Sergey V Razin

**Affiliations:** 1 Institute of Gene Biology of the Russian Academy of Sciences, 34/5 Vavilov str., 119334, Moscow, Russia; 2 Faculty of Biology, M.V. Lomonosov Moscow State University, 1/12 Leninskie gory, 119992, Moscow, Russia; 3 University of Oslo, Center for Medical Studies in Russia, Moscow, Russia; 4 LIA 1066 French-Russian joint cancer research laboratory

**Keywords:** β-globin genes, Active chromatin hub, Insulator, Chicken RBCs

## Abstract

**Background:**

The β-globin gene domains of vertebrate animals constitute popular models for studying the regulation of eukaryotic gene transcription. It has previously been shown that in the mouse the developmental switching of globin gene expression correlates with the reconfiguration of an active chromatin hub (ACH), a complex of promoters of transcribed genes with distant regulatory elements. Although it is likely that observations made in the mouse β-globin gene domain are also relevant for this locus in other species, the validity of this supposition still lacks direct experimental evidence. Here, we have studied the spatial organization of the chicken β-globin gene domain. This domain is of particular interest because it represents the perfect example of the so-called ‘strong’ tissue-specific gene domain flanked by insulators, which delimit the area of preferential sensitivity to DNase I in erythroid cells.

**Results:**

Using chromosome conformation capture (3C), we have compared the spatial configuration of the β-globin gene domain in chicken red blood cells (RBCs) expressing embryonic (3-day-old RBCs) and adult (9-day-old RBCs) β-globin genes. In contrast to observations made in the mouse model, we found that in the chicken, the early embryonic β-globin gene, *Ε*, did not interact with the locus control region in RBCs of embryonic lineage (3-day RBCs), where this gene is actively transcribed. In contrast to the mouse model, a strong interaction of the promoter of another embryonic β-globin gene, *ρ*, with the promoter of the adult β-globin gene, *β*^*A*^, was observed in RBCs from both 3-day and 9-day chicken embryos. Finally, we have demonstrated that insulators flanking the chicken β-globin gene domain from the upstream and from the downstream interact with each other, which places the area characterized by lineage-specific sensitivity to DNase I in a separate chromatin loop.

**Conclusions:**

Taken together, our results strongly support the ACH model but show that within a domain of tissue-specific genes, the active status of a promoter does not necessarily correlate with the recruitment of this promoter to the ACH.

## Background

The domain of chicken beta-globin genes is located on chromosome 1 and has a length of approximately 33 kb. It includes a cluster of four beta-globin genes: *ρ* (*HBG1*), *β*^*H*^ (*HBE1*), *β*^*A*^ (*HBG2*) and *Ε* (*HBE*) and several distant regulatory regions, which are marked with sites of hypersensitivity to DNase I (HS) and are necessary for the regulation of transcription, replication and chromatin status of the domain [1,2]. The locus control region of the domain (LCR) is located upstream of the embryonic β-globin gene *ρ* and is composed of three blocks co-localizing with the erythroid cell-specific HSs 1 to 3 [3,4]. A constitutive HS4 located upstream of the LCR marks the position of the well-studied CTCF-dependent (CTCF ‐ Ð¡Ð¡Ð¡TC-binding protein factor) insulator [5-9]. The CTCF-dependent enhancer-blocking element is also located at the downstream end of the domain in the area marked by the so-called 3′ constitutive HS [10,11]. In addition to the LCR, the domain also possesses an internal enhancer (β/Ε-enhancer), which is located between the genes *β*^*A*^ and *Ε* and is believed to contribute to the control of the activity of both genes [12-14]. The partitioning of tasks between the LCR and the β/Ε-enhancer is not quite clear. The results of experiments with transgenic mice carrying the chicken β-globin gene domain [4] suggested that the expression of one of the embryonic genes (*ρ*) is controlled by the LCR, while the expression of the other embryonic gene (*Ε*) is controlled by the β/Ε-enhancer. In addition, both regulatory elements appeared to be necessary for the correct developmental expression of the adult β-globin genes [3]. The domain of the chicken β-globin gene represents one of the best-studied genomic areas in higher eukaryotes [15]. This domain possesses all of the characteristic features of the so-called closed or strong chromatin domains [16,17], which change the pattern of sensitivity to DNase I in a cell lineage-specific fashion. Detailed studies of the distribution of different histone modifications along the domain have contributed much to the present knowledge about the histone code [18,19]. Nevertheless, the mechanisms of transcriptional switching of chicken β-globin gene expression remain poorly understood [15]. The developmental switching of globin gene transcription is typical for all vertebrate organisms [2]. In chickens, the first molecules of hemaglobin appear in embryonic erythroblasts 32 h after fertilization [20]. The beta-chains of the hemaglobin represent the products of genes *ρ* and *Ε*, which are transcribed from the 2^nd^ to the 5^th^ day of the embryonic development. After the 5^th^ day a new population of erythroid cells expressing the genes of fetal and adult globins *β*^*Н*^ and *β*^*A*^ appears. These cells gradually replace the embryonic-type erythroblasts that express genes 1*ρ* and *Ε*[21]. In erythroblasts of adult lineage, the transcription of embryonic β-type globin genes is repressed.

**Figure 1:**
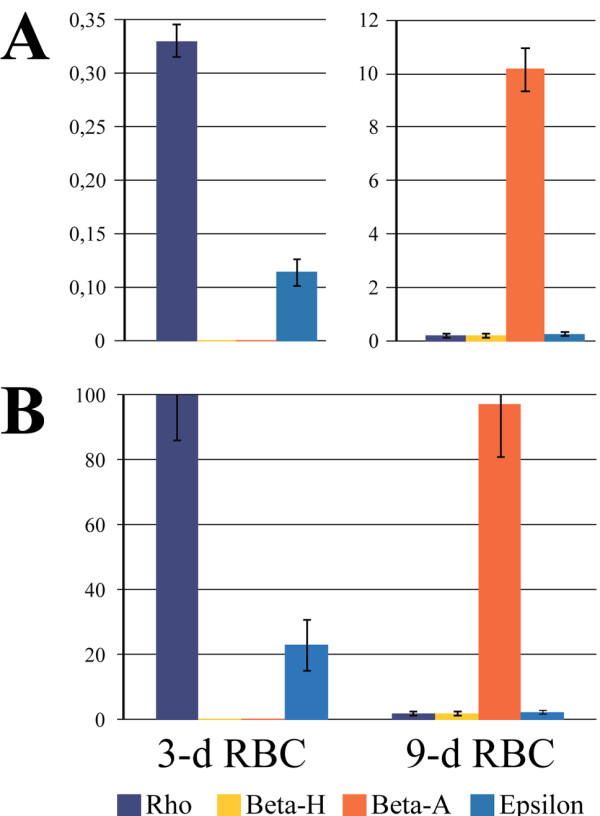
**Analysis of β-globin mRNA levels in chicken 3-day and 9-day embryonic red blood cells.** The vertical axis shows the relative amounts of β-globin gene primary transcripts, as determined by RT-qPCR analysis of RNA samples prepared from the blood of 3- and 9-day old chicken embryos. **(A)** Normalization of data to the level of β-actin mRNA. **(B)** Normalization of data to the amount of cells used for RNA preparation. The amounts of β-globin gene transcripts detected in different RNA samples were normalized to the number of cells used for preparation of these RNA samples. The amount of the most abundant transcript (ρ-transcript in 3-day RBCs) was set as 100 relative units and the other data were normalized to this value. The error bars represent the S.E.M. for two independent experiments.

Recent studies of the spatial configuration of the mouse and human β-globin gene domain suggest that in erythroid cells, all regulatory elements of the domain become assembled into a common activation complex (the ACH) where the promoters of different globin genes are recruited in a developmental stage-specific fashion [22-24].

In the present work, we have analyzed the spatial configuration of the chicken β-globin gene domain in circulating red blood cells (RBCs) both before (3-day RBCs) and after (9-day RBCs) the β-globin switching. Using the quantitative chromosome conformation capture assay (3C-qPCR) [25,26], we have demonstrated that the switching of chicken β-globin gene expression correlates with prominent changes in the spatial configuration of the domain. However, the observed spectrum of changes does not perfectly fit the simplistic model that postulates that only the transcriptionally active genes are recruited to the ACH.

## Results

### Measurement of β-globin mRNA levels in red blood cells of 3- and 9-day-old chicken embryos

We first estimated the relative levels of primary transcripts of each of the β-globin genes in RBCs (red blood cells) of 3-and 9-day-old chicken embryos using reverse transcription with subsequent TaqMan™ real-time PCR. To exclude the spliced mRNA from this analysis, we used test amplicons that spanned exon-intron junctions. The results obtained (Figure 1) were consistent with the previously reported data [1,21]. In 3-day RBCs, only embryonic *Ε* and *ρ* genes were actively transcribed, with the level of ρ transcripts being approximately 4.5-fold higher than the level of Ε transcripts. In 9-day RBCs, transcripts of all four β-globin genes were detected, and as expected, the transcript of the *β*^*A*^ gene was the most abundant. The presence of small amounts of *Ε* and *ρ* gene transcripts in 9-day RBCs is again consistent with a previous observation that approximately 10% of chicken embryonic RBCs express Ε- and ρ-globin even on the 10^th^ day of development [1].

**Figure 2:**
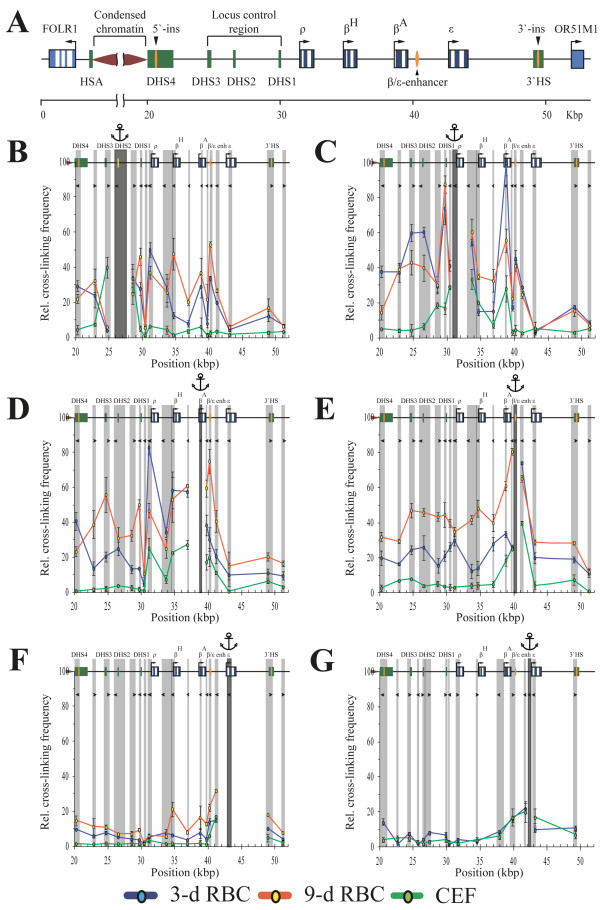
**MboI and NlaIII q3C analysis of the chicken β-globin gene domain. (A)** Map showing the positions of genes and known regulatory elements of the chicken β-globin gene domain. The open boxes indicate genes, the blue lines represent exons, and the arrows indicate the direction of transcription. The closed green boxes show HSs. Upstream insulator, downstream enhancer-blocking elements and the β/Ε enhancer are shown by orange ellipses. *FOLR1* - gene encoding folate receptor, transcribed in earlier erythroid progenitors; HAS - tissue-specific erythroid DNase I-hypersensitive site; HS1-3 - locus control region (LCR) of β-globin gene domain; *OR51M1* - open reading frame for odorant receptor. The map was made using nucleotide sequences NW_001471556 and L17432.1 [GenBank:NW_001471556, GenBank:L17432.1]. **(B-G)** The relative frequencies of crosslinking of the anchor fragments containing the HS2 **(B)**, ρ-promoter **(C)**, upstream portion of gene *β*^*A*^**(D)**, β/Ε-enhancer **(E)**, upstream portion of gene *Ε***(F)** and Ε-promoter **(G)**. **(B-F)** show results of MboI 3C analysis and (G) shows the results of NlaIII 3C analysis. The dark grey rectangle in each panel indicates the anchor DNA fragment, and the light grey rectangles indicate the test fragments. The white rectangles indicate the restriction fragments that were not analyzed. The borders between neighboring fragments are indicated by dark grey lines. The tailless arrows represent the primers used for q3C analysis. The positions of test fragments on the restriction map are plotted on the x-axis. The cross-linking frequencies are plotted on the y-axis; the highest cross-linking frequency observed was set as 100 relative units (the cross-linking frequency between the fragment containing the ρ-promoter and the fragment containing the *β*^*A*^ gene in 3-day RBCs for MboI 3C analysis, and the cross-linking frequency between the fragment containing HS2 and the fragment containing HS1 in 3-day RBCs for NlaIII 3C analysis (data not shown)). The results of 3C analysis for 3-day RBCs, 9-day RBCs and CEF are shown by the blue, red and green lines, respectively. The error bars represent the S.E.M. for three (B-F) or two (G) independent experiments.

To be able to compare the relative levels of transcription of different globin genes in 3-day and 9-day RBCs, the experimentally detected amounts of globin gene primary transcripts were normalized to the experimentally detected amount of β-actin mRNA (Figure 1A). However, it is worth mentioning that the total amount of RNA routinely extracted from the same number of 3-day and 9-day RBCs was much higher in the case of 3-day RBCs (approximately 50 μg and 1.7 μg from 10 million 3-day and 9-day RBCs, respectively). Consequently, the amounts of mRNAs of housekeeping genes differ drastically in samples prepared from the same number of 3-day and 9-day RBCs. For this reason, normalization to the amount of cells (3-day and 9-day RBCs) used for the extraction of RNA (Figure 1B) appears to be more appropriate for comparison of the absolute levels of globin gene transcription in these cells. Indeed, using this normalization one can see that total amounts of β-globin gene transcripts are comparable in 3-day and 9-day RBCs while the spectrums of transcribed β-globin genes are different.

### Interaction of the locus control region and β/Ε enhancer with promoters of β-globin genes in red blood cells of 3-day and 9-day chicken embryos

The quantitative chromosome conformation capture (3C-qPCR) assay was used to investigate the spatial organization of the chicken β-globin gene domain in RBCs of 3- and 9-day-old embryos. It is well known that the accurate analysis and interpretation of raw 3C data is not a trivial task [27], and it can be difficult to distinguish the relevant chromatin interactions from background and cross-linking artifacts. One problem in the interpretation of the 3C data is that neighboring restriction fragments are always ligated to a certain extent due to cross-links between nucleosomal proteins, even if these fragments are not involved in the assembly of a chromatin hub. To discriminate the functionally relevant interactions from the background cross-linking, we performed the 3C analysis of the β-globin gene domain in chicken embryonic fibroblasts (CEF). These cells do not express globin genes. Thus, there are good reasons to expect that the domain in these cells does not adopt any functionally relevant spatial configuration.

In the main set of 3C experiments, we used MboI restriction enzyme digestion to cleave the DNA before the proximity ligation. This enzyme cuts the β-globin gene domain into a number of relatively small fragments, such that the promoters of β-globin genes and regulatory elements were located in different restriction fragments separated by several cleavage sites. It was thus possible to independently analyze all of these regulatory elements. In some cases, however, it was not possible to place the primers and TaqMan™ probes exactly on the regulatory element under study due to the small size of the corresponding restriction fragment or the presence of repetitive sequences. In these cases, the primers and TaqMan™ probes were placed at the adjacent restriction fragments. For example, for *β*^*A*^ and *Ε* genes, the probes were placed at the fragments located just downstream of the promoters.

We have first fixed the anchor at the restriction fragment containing HS2 (a part of β-globin domain LCR). In the 3-day RBCs, this anchor was shown to interact preferentially with the fragments containing the promoter of the embryonic gene *ρ*, the adult gene *β*^*A*^ and the β/Ε-enhancer (Figure 2B, blue curve). In addition, an elevated frequency of interaction of the HS2 with the upstream and the downstream insulators of the β-globin gene domain was observed. Interestingly, no interaction of the HS2 with the embryonic gene *Ε* was detected. As expected, no interaction between any of the functional elements studied was detected in embryonic fibroblasts (Figure 2B, green curve). In 9-day RBCs the frequency of interactions of the HS2 with the *β*^*A*^ gene increased. In addition, a strong interaction between the HS2 and the promoter of *β*^*H*^ gene was established (Figure 2B, red curve). The validity of the observations made with the anchor placed at HS2 was confirmed in experiments with the anchor placed on the promoter of the *ρ* gene (Figure 2C), the upstream portion of the *β*^A^ gene (Figure 2D), the upstream portion of the *Ε* gene (Figure 2F) and the β/Ε-enhancer (Figure 2E). The anchor placed on the promoter of the *ρ* gene demonstrated strong interaction frequencies with the HSs 1‐3 of the LCR in 3-day RBCs (Figure 2C, blue curve). The frequency of interaction of this anchor with the HS1 remained about the same in 9-day RBCs while the frequencies of interaction with the HSs 2 and 3 decreased (Figure 2C, red curve). Unexpectedly, in 3-day RBCs the promoter of the *ρ* gene demonstrated a strong interaction with the upstream region of the *β*^*A*^ gene. The frequency of this interaction decreased in 9-day RBCs but still remained quite prominent (Figure 2C, blue and red curves). The interaction between *ρ* and *β*^*A*^ genes was also observed in embryonic fibroblasts (Figure 2C, green curve). It may thus reflect some internal feature of the β-globin gene domain spatial organization that is not related to the transcriptional status of the domain. The interaction between the *β*^*A*^ and *ρ* gene promoters observed in the experiment with the anchor placed on the *ρ* gene promoter was confirmed in the reciprocal experiment with the anchor placed on the upstream part of the *β*^A^ gene (Figure 2D, all curves). No interaction of the *ρ* gene promoter with the promoter of the minor adult *β*^*H*^ gene was observed in 3-day RBC (Figure 2C, blue curve). In contrast, in the same cells (3-day RBC) the upstream region of the *β*^*A*^ gene demonstrated strong interactions with both the *ρ* and *β*^*H*^ gene promoters (Figure 2D, blue curve). As expected, the upstream region of the *β*^*A*^ gene demonstrated a strong interaction with the HSs 1 to 3 of the LCR in 9-day RBCs, but not in 3-day RBCs (Figure 2D, red and blue curves). A strong interaction of the *β*^*A*^ gene with the β/Ε enhancer was also observed in 9-day but not in 3-day RBCs (Figure 2D, red and blue curves). Although in this particular case the anchor (the upstream part of the *β*^*A*^ gene) and the test-fragment (β/Ε enhancer) are located close to each other, the much higher interaction frequency observed in 9-day RBCs comparing to 3-day RBCs and CEF argues for the existence of functionally relevant interaction between the *β*^*A*^ gene and the β/Ε enhancer in 9-day RBCs. This conclusion is supported by the results of a reciprocal experiment with the anchor placed on the β/Ε enhancer. Again, in 9-day RBCs the frequency of interaction of the β/Ε enhancer with the upstream part of the *β*^*A*^ gene was much higher in 9-day RBCs than in 3-day RBCs and CEF (Figure 2E). The interaction of the β/Ε enhancer with the three HSs of the LCR and the promoter of *ρ* gene was observed in both 3-day and 9-day RBCs, but not in CEF. On the other hand, interaction of the β/Ε enhancer with the promoter of gene *β*^H^ was observed only in 9-day RBCs (Figure 2E, red curve). No interaction of the β/Ε enhancer with the gene *Ε* was observed in either of the cell lines studied (Figure 2E). In full accord with this observation, the anchor placed on the upstream area of the gene *Ε* showed no interaction with any of the test fragments studied in either erythroid or non-erythroid cells (Figure 2F). Thus it appears that the *Ε* gene does not interact with any of the remote regulatory elements of the domain. To verify this interesting observation, we performed 3C experiments using digestion with another restriction enzyme, NlaIII. Distribution of NlaIII recognition sites along the β-globin gene domain allows fixation of the anchor exactly on the promoter of the gene *Ε*. Using this anchor we performed 3C analysis of the β-globin domain configuration in 3-day RBCs and in CEF. Again, no interaction of the *Ε* gene promoter with any of the remote regulatory elements was observed (Figure 2G). As a matter of fact, the frequencies of interaction of the *Ε* gene promoter with all tested fragments of the β-globin gene domain were very similar in 3-day RBCs that express the *Ε* gene and in embryonic fibroblasts that do not express globin genes at all.

### Interaction between the upstream and the downstream insulators of the β-globin gene domain

**Figure 3:**
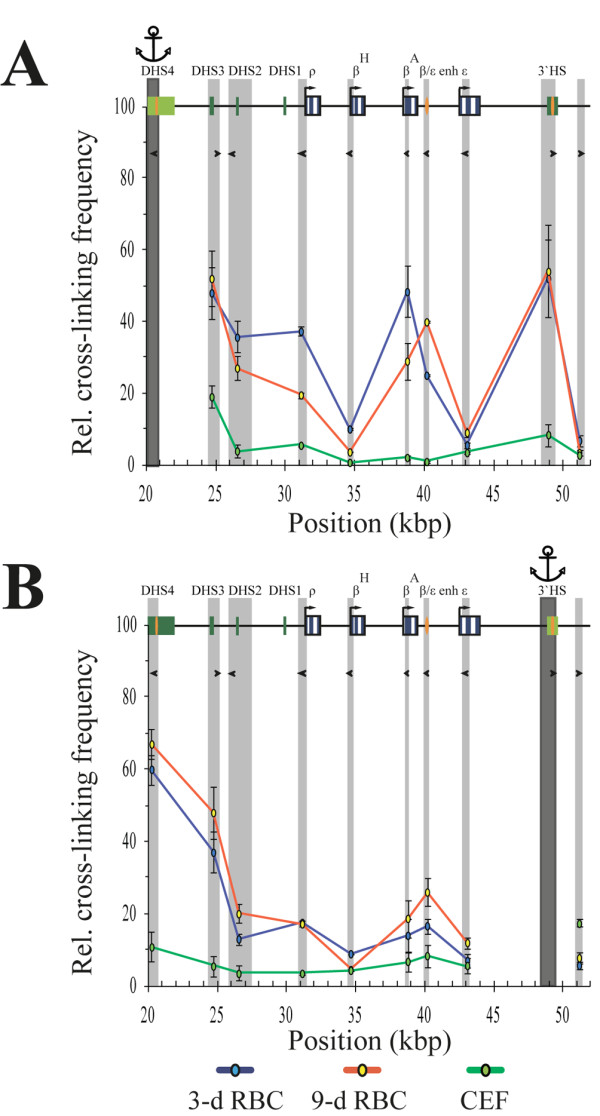
**MboI q3C analysis of spatial interactions of β-globin boundary insulators.** The relative frequencies of cross-linking of the anchor fragments containing the 5′-insulator **(A)** and 3′-insulator **(B)** of the chicken β-globin gene domain. All designations are as described in Figure 2.

Figure 3 demonstrates the results of 3C experiments with the anchors placed on either upstream or downstream insulators of the β-globin gene domain. It is evident that in both 3-day and 9-day RBCs, the insulators strongly interact with each other, thus forming a loop harboring the entire β-globin gene domain. In addition, in 9-day RBCs, both insulators demonstrate an interaction with the β/Ε enhancer, while in 3-day RBCs, the upstream insulator interacts with the *β*^*A*^ gene. Finally, in both the 3-day and the 9-day RBCs, both insulators show some interaction with the LCR. In embryonic fibroblasts neither the interaction between the upstream and the downstream insulators of the β-globin gene domain nor an interaction of these insulators with any of the internal fragments of the domain was observed (Figure 3A/B, green curves).

## Discussion

The current model of the enhancer action suggests that enhancers directly interact with target promoters [23,28-30]. As a result, an assembly of regulatory elements, called the active chromatin hub (ACH), forms. One or several enhancers present in the ACH may control the activity of one or several promoters recruited to the ACH [23,29]. The transcriptional switching of murine β-globin gene expression correlates with a reconfiguration of the ACH [24]. The aim of the present study was to determine whether the developmental switching of chicken β-globin gene transcription depends on the spatial reconfiguration of the β-globin gene domain. The study of the chicken β-globin gene domain is particularly interesting for two reasons. First, this domain is the best example of the so-called closed chromatin domains [17], as the upstream and the downstream insulators perfectly delimit the area of preferential sensitivity to DNase I in erythroid cells [31], which is not the case in mouse and human β-globin gene domains [32,33]. Second, the chicken β-globin gene domain contains an internal enhancer element in addition to the upstream locus control region [12]. Once again, this is not the case in the mouse or human. Hence, the regulation of chicken β-globin gene expression may differ from regulation of β-globin gene expression in the mouse and human.

The results of the present study showed that in erythroid cells there is a clear interaction of the upstream and the downstream insulators of the chicken β-globin gene domain. As a result, the domain should be organized in a closed loop. Although we have not studied the mechanism of this loop formation, it is very probable that CTCF is involved, as both insulators possess CTCF-binding sites [9,10] and it was shown that CTCF might mediate an interaction between distant genomic elements [34,35]. It is also likely that the β-globin loop ends are fixed at the nuclear matrix, as the 5′ insulator of the chicken β-globin gene domain has been reported to interact with the nuclear matrix [36]. This type of spatial organization perfectly explains the fact that in the chicken β-globin gene domain, the area demonstrating the cell-lineage-specific sensitivity to DNase I perfectly co-localizes with the genomic segment flanked by insulators. In agreement with this model, the interaction between the upstream and the downstream insulators of the chicken β-globin gene domain was not observed in non-erythroid cells where the domain is not preferentially sensitive to DNase I [37]. In should be mentioned, however, that in mouse and human the insulators flanking the β-globin gene domain from the upstream and the downstream also interact with each other [22,24,34], although the chromatin loop established by this interaction does not correspond to the region of preferential sensitivity to DNase I [32].

The most interesting feature of the spatial organization of the chicken β-globin gene domain is that the embryonic β-globin gene Ε interacts with neither the LCR nor the β/Ε enhancer. This does not exclude the possibility that the β/Ε enhancer located at a short distance upstream of the *Ε* gene can activate this gene promoter by some tracking mechanism. However, an activation effect of the LCR on *Ε* gene transcription seems very improbable. In this respect, it is of interest that in the chicken α-globin gene domain, the embryonic gene *π* also does not interact with any of the known distant regulatory elements of the domain [38]. It is thus likely that in the chicken, both α-type and β-type early embryonic globin genes possess an autonomous regulatory system.

**Figure 4:**
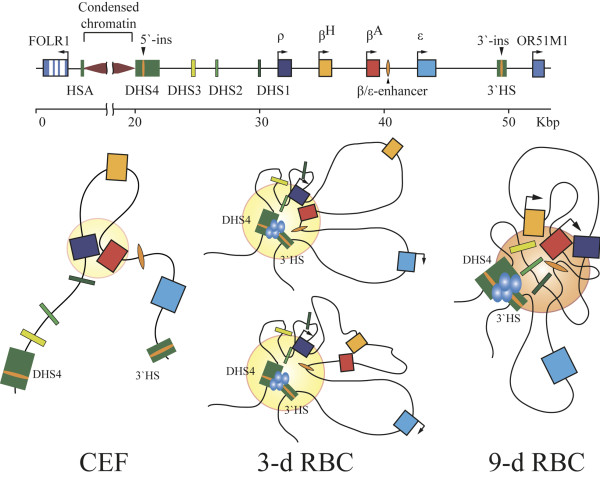
**β-globin active chromatin hubs in the nuclei of 3-day and 9-day red blood cells of chicken embryos.** The filled disks designate ACH, and the blue ellipses show protein complexes assembled on the boundary insulators.

In regard to the composition of the β-globin domain ACH in 3-day and 9-day RBCs, we noted several unexpected features. First of all, the HS2 of LCR shows a significant frequency of interaction with the promoter of the *β*^*A*^ gene already in 3-day RBCs where the level of this gene transcription is very low. Still it is detectable. Furthermore, it was reported that the *β*^*A*^ gene is transcribed to some extend even in 2-day RBCs [39]. Hence, the interaction of the *β*^*A*^ gene with the LCR in 3-day RBCs may be functionally relevant. On the other hand, we observed a strong interaction between the promoters of the *ρ* and *β*^*A*^ genes, which was especially prominent in 3-day RBCs in which the level of the *β*^*A*^ gene transcription is very low. The functional significance of this interaction is not clear at the moment. The fact that it was detected also in non-erythroid cells suggests that it may reflect some inherent feature of the β-globin gene domain spatial organization that is not directly related to the assembly of ACH. It is of interest that despite the above-mentioned interaction between the *ρ* and *β*^*A*^ promoters, the levels of interaction of these promoters with the LCR-HS2 differ significantly in 3-day RBCs (where the promoter of gene *ρ* interacts preferentially with the LCR-HS2) and 9-day RBCs (where the promoter of gene *β*^*A*^ interacts preferentially with the LCR-HS2). Thus, it is likely that the fine structure of the β-globin ACH is quite different in 3-day and 9-day RBCs. We suggest that in 3-day RBCs, the promoter of gene *ρ* directly interacts with the LCR while the promoter of gene *β*^*A*^ is passively pulled to the complex due to the interaction of *β*^*A*^ and *ρ*. In 9-day RBCs the situation is reversed. Thus, in spite of interaction with each other, the genes *ρ* and *β*^*A*^ demonstrate developmental stage-specific association with the LCR which correlates with the expression status of these genes. Even more evident developmental stage-specific recruitment to the ACH shows the promoter of the minor adult β-type globin gene *β*^*H*^. The promoter of this gene demonstrates either no interaction or a strong interaction with the LCR at days 3 and 9 of the embryo development, respectively. Finally, it should be mentioned that in both 3-day and 9-day RBCs, a significant level of interaction between the *β*^*A*^ and *β*^*H*^ promoters was observed. Taking into consideration the fact that the anchors placed on the LCR-HS2 and *ρ* gene promoter demonstrated no interaction with *β*^*H*^ and a strong interaction with *β*^*A*^ in 3-day RBCs, one must assume that at this developmental stage, the promoters of the two adult β-type globin genes can either interact with each other outside the ACH or the β^A^ promoter can be recruited to the ACH *via* interaction with the *ρ* gene promoter. The two alternative configurations of the β-globin domain ACH in 3-day RBCs are shown in Figure 4.

In 9-day RBCs, the level of mutual interaction of most of the regulatory elements of the β-globin gene domain increases compared to the levels in 3-day RBCs. These observations may reflect more compact packaging of the domain as a whole. Thus, it should be noted that 9-day RBCs differ drastically from the 3-day RBCs in the total amount of RNA, including rRNA, present in the cell (see the first section of the results). Evidently, the 9-day RBCs are less metabolically active compared to 3-day RBCs. In other words, they possess certain features of mature chicken erythrocytes, which have non-active nuclei. Nevertheless, the globin genes are still transcribed in the majority of 9-day RBCs [40]. Thus, the concept that the apparently more compact spatial organization of the β-globin gene domain in 9-day RBCs may simply reflect the presence of a substantial portion of mature erythrocytes in the analyzed cell population would not be correct. Rather, the β-globin gene domain suffers a certain amount of pressure because it is a part of the chromosomal territory that gradually acquires a more compact configuration. Regardless, it remains active and possesses certain features of an active chromatin domain, including a preferential sensitivity to DNase I, even in the compact nuclei of mature chicken erythrocytes [41]. The spatial isolation of this domain from the surrounding chromatin by the formation of a distinct chromatin loop *via* interaction of the flanking insulators may be of major importance.

## Conclusions

In this work, we have analyzed the spatial configuration of the chicken β-globin gene domain in red blood cells of embryonic and adult lineages. The obtained results strongly support the active chromatin hub model, as it has been found that distant regulatory elements located upstream of the β-globin gene cluster form a complex with the β/Ε enhancer located in the downstream portion of the cluster. Promoters of all β-globin genes, with the exception of embryonic gene *Ε*, are recruited to this complex, demonstrating a specificity of interaction with the LCR in respect to the developmental stage. The embryonic β-globin gene *Ε* does not interact with any of the distal regulatory elements; thus, it is likely to possess an autonomous regulatory system. In both 3-day and 9-day RBCs the upstream and the downstream insulators of the chicken β-gene domain interact with each other, thus placing the domain in a separate chromatin loop. This may explain the fact that in chickens, the borders of erythroid cell-specific domain with preferential sensitivity to DNase I perfectly co-localize with the borders of β-globin gene domain marked by insulators.

## Methods

### Real-time reverse transcriptase polymerase chain reaction (RT-PCR)

Chicken red blood cells (RBCs) were collected from 3- and 9-day embryos and directly used for analysis. Chicken embryonic fibroblasts (CEFs) were isolated from 9-day embryos according to the standard protocol [42] and grown in DMEM supplemented with 8% FBS and 2% chicken serum. Total RNA was extracted using the TRIzol™ Reagent (Invitrogen, Life Technologies, Carlsbad, CA, USA) and was treated with DNase I (Fermentas, Vilnius, Lithuania) to remove residual DNA. RNA (0.5 μg) was reverse transcribed in a total volume of 20 μl for 1 h at 42°C using 0.2 μg of random hexamers and 200 U of reverse transcriptase M-MuLV (Fermentas, Vilnius, Lithuania) in the presence of 20 U of ribonuclease inhibitor (Fermentas, Vilnius, Lithuania). The resulting cDNA was analyzed by TaqMan™ real-time PCR. For β-globin genes, the amplicons were designed in such a way that they spanned intron-exon junctions. For β-actin gene the amplicon was within an exon. The PCR primers and TaqMan™ probes are presented in Table 1.

**Table 1:** Primers and TaqMan™ probes used for analysis of β-globin primary transcripts and β-actin mRNA levels

**Primers**	
Rho F	CAGAACACTGGGGTTTGGCT
Rho R	GGCCAGCACAATGATGAGG
BetaH F	CCAGAGGTTCTTTGCGTCC
BetaH R	CTCAGTCCTATAACTCCTGCCTA
BetaA F	TACACTTCTGTTCCCCATCTCTC
BetaA R	CCAAAGGACGCAAAGAACC
Epsilon F	TAGGCAAAGAGTGAGCATTCG
Epsilon R	GCCAGGACGATGATCAGGAT
Beta-actin F	TGGACTCTGGTGATGGTGT
Beta-actin R	TCTCTCTCGGCTGTGGTGG
**TaqMan™ probes**	
Rho	FAM-CTCCACACC(T-BHQ1)TGCTTACCCCATTG-PO4
BetaH	FAM-ACAACA(T-BHQ1)CAAGAAGAGCTTTGCCCAG-PO4
BetaA	FAM-CTGATCGTC(T-BHQ`)ACCCCTGGACCCAGA-PO4
Epsilon	FAM-CTGTGCTTGG(T-BHQ1)GGGAAGAGGGGAT-PO4
Beta-actin	FAM-TCTATGAAGGC(T-BHQ1)ACGCCCTCCCCC-PO4

5′-3′ sequences are presented. Sequences [GenBank:NW_001471556] for β-globin and [GenBank:NC_006101] for β-actin primers were used.

### 3C analysis

The 3C analysis was performed as described [43] with minor modifications. Briefly, 7.5×10^7^ cells were fixed in DMEM/serum with 2% formaldehyde for 10 min at room temperature. The reaction was stopped by adding glycine to a concentration of 0.125 M and placing the samples on ice. Cross-linked cells were washed with cold PBS and lysed by incubation for 10 min in ice-cold lysis buffer (10 mM Tris (pH 8.0), 10 mM NaCl, 0.2% NP-40, protease inhibitor cocktail R1321 (Fermentas, Vilnius, Lithuania)). The nuclei were harvested, and aliquots containing 3.75×10^7^ nuclei were frozen in liquid nitrogen and stored at ‐70°C.

Approximately 1.25×10^7^ nuclei were suspended in 0.25 ml of an appropriate 1.2x restriction buffer, SDS was added to a final concentration of 0.3%, and the solution was incubated with shaking at 37°C for 1 h. The solution was then diluted 2-fold with the same restriction buffer, and Triton X-100 was added to a final concentration of 1.8% (to sequester the SDS) followed by incubation at the same conditions for another hour. After incubation, 400 U of MboI (NEB, Hitchin, UK) or NlaIII (Fermentas, Vilnius, Lithuania) was added, and the digestion was performed for 20 h at 37°C with continuous shaking. The reaction was terminated by the addition of SDS to a final concentration of 1.3% and incubation at 65°C for 20 min. The solution was diluted by adding 7 ml of 1x ligation buffer (50 mM Tris (pH 7.5), 10 mM MgCl_2_, 10 mM DTT, 1 mM ATP). Triton X-100 was added to a final concentration of 1%, and the solution was incubated at 37°C for 1 h while shaking. T4 DNA Ligase (100 U, Fermentas, Vilnius, Lithuania) was then added, and DNA was ligated at 14-16°C for 5 h followed by further incubation at room temperature for 30 min with slow agitation. Cross-links were reversed by incubation at 65°C for 16 h in the presence of 500 μg of Proteinase K (Sigma). After cross-link reversion, 300 μg of RNase A was added, and the RNA was digested at 37°C for 45 min. DNA was purified by extraction with phenol (pH 8.0), followed by precipitation with ethanol in the presence of 120 μg of glycogen (Fermentas, Vilnius, Lithuania).

The efficacy of restriction enzyme digestion in 3C reactions was checked using PCR stop analysis and was found to be more than 80% for different sites throughout the locus under study (data not shown).

To generate a random ligation control, 8 μg of a bacterial artificial chromosome containing the chicken β-globin gene domain along with the flanking areas [CHORI BACPAC Resources Center:TAM32-28 J3] was digested with Sau3A or NlaIII and then ligated at a high DNA concentration.

The TaqMan™ real-time PCR technology was employed to analyze the ligation products. Primers and TaqMan™ probes for PCR were designed using the DNA sequence [GenBank: NW_001471556] and Primer Premier 5 computer software (PREMIER Biosoft International, Palo Alto, CA, USA). The sequences of the primers and TaqMan™ probes are present in Table 2. A real-time PCR reaction in a total volume of 20 μl included 20‐100 ng of a 3C DNA template or the same amount of digested and re-ligated chicken genomic DNA along with 3, 0.3, 0,003 or 0,0003 ng of the control BAC template, 1x PCR buffer (50 mM Tris (pH 8.6), 50 mM KCl, 1.5 mM MgCl_2_, 0.1% Tween 20), 10 pmol of each primer, 5 pmol of a TaqMan™ probe (5′-FAM dye, inside BHQ-1quencher), 4 nmol of each dNTP and 0.75 U of Hot start Taq DNA polymerase (Sibenzyme, Novosibirsk, Russia). The reaction was performed as follows: initial denaturation for 5 min at 94°C, 50 cycles of 15 s at 94°C and 60 s at 60°C followed by reading the plate. All real-time PCR component reactions were aliquoted and stored at ‐70°C until required for PCR with each new primer pair.

**Table 2:** Primers and TaqMan™ probes used for 3C analysis

	**Primers for MboI 3C analysis**
HS4 (anchor)	GAGAATCGGGTGCAGGCTT
HS4 -|- HS3	GCGTGGATAGAAGTAAACTGGAA
HS3	GCAGTCCCAGGTTGCACAA
HS2 (anchor)	CACCGTCAGCATACATTGCT
HS2 -|- HS1	CAGGACCACCGTTACCCAT
HS1	TCAGGAACCGTGGGTGTGT
HS1 -|- Rho	ACAACTCCAAGGGATGAAGC
Rho (anchor)	AGGGAGACTACGAATGCTGC
Rho -|- betaH	AATGGAAAGGTTCTGTCTCAGC
BetaH	CTGAGCCCCACCCTGATG
BetaH -|- BetaA	ACTATGTCCATCCCAGCTCCA
BetaA (anchor)	AGCCCCACTGCCATCCTT
BetaA -|- Beta/eps enh	TCACGGAGCACCTACAACCA
Beta/eps enh (anchor)	CTATCAAGACTTGCACAGACCTT
Beta/eps enh -|- Epsilon	AGTGATTCTATGGAGGAAGGCA
Epsilon (anchor)	GCGACAAGTTGCACGTGG
3′HS (anchor)	GGGCATTTCTACAGAGAGCAA
Downstream to 3′HS	CCACATCTGCCAAGAGCCT
ERCC3 test	CAAATCTGACAGCCGCTTAGA
ERCC3 (anchor)	CTCGATGAAGTACATACTATTCCTG
	**Primers for NlaIII 3C analysis**
HS4	GTGGATTGAAGGATGCTGAG
HS4 -|- HS3	CCAAAGGACCCCAGGACTA
HS3	TGACACAGCACAAAAGTGAGC
HS3 -|- HS2	AGCAATGTATGCTGACGGTG
HS2	AAATACATCCCATCCTGGTGTTC
HS2 -|- HS1	CAGGAAAAGCCTTACTTCAAATG
HS1	CCGCTGCTCAGTGTCACAC
HS1 -|- Rho	CGAAGTAGGGAACCTCAACA
Rho	AGGAGGGGAGTAAAAGGGAG
BetaH	CCCTGAACGATGAGTAGGAAGT
BetaA	CAGCTCTGCAGCTCTATACCG
Beta/eps enh	TTAATCCTCCCTGGATCATTTC
Beta/eps enh -|- Epsilon	GCTCTGCTTACATTCACATCCA
Epsilon (anchor)	AGCGCTGCCCTACCAGAC
Epsilon -|- 3′HS	AGCAGCCTGCAGAGGGAT
3′HS	GGGCATTTCTACAGAGAGCAA
ERCC3 test	CAAATCTGACAGCCGCTTAGA
ERCC3 (anchor)	CTCGATGAAGTACATACTATTCCTG
	**TaqMan™ probes for MboI 3C analysis**
HS4	FAM-ATTCCCTGTCC(T-BHQ1)GTCCCACAGCTGT-PO4
HS2	FAM-AATTTGTGA(T-BHQ1)GGACTTCAGGCCCAT-PO4
Rho	FAM-AAAGGAGCTGAC(T-BHQ1)CATGCTAGCCCAG-PO4
BetaA	FAM-TTTGCCTCC(T-BHQ1)TTGGGAACCTCTCC-PO4
Beta/eps enh	FAM-CAGTTCTCCGAG(T-BHQ1)CTGACTTTGGAGTCT-PO4
Epsilon	FAM-TTTGCCTCC(T-BHQ1)TTGGGAACCTCTCC-PO4
3′HS	FAM-CAGTTCTCCGAG(T-BHQ1)CTGACTTTGGAGTCT-PO4
ERCC3	FAM-TGGAGTGGCT(T-BHQ1)AAAAGTCGAGAGTGG-PO4
	**TaqMan™ probes for NlaIII 3C analysis**
HS2	FAM-AAGTG(T-BHQ1)CCCAGCTCCTGTTCTCCCAC-PO4
Epsilon	FAM-CCTTTCTC(T-BHQ1)GAAAAGGGGCCCAGGT-PO4
ERCC3	FAM-TGGAGTGGCT(T-BHQ1)AAAAGTCGAGAGTGG-PO4

5′-3′ sequences are presented. Sequences [GenBank:NW_001471556] for β-globin and [GenBank:NC_006094] for ERCC3 primers were used. Symbol ”-|-”designates primers located in linkers between functional elements within the domain.

To account for the qualitative and quantitative differences of independently prepared 3C samples, the qPCR data for each sample were normalized to the results obtained by using primers for the housekeeping gene, *ERCC3* (encodes the ATP-dependent DNA-helicase, a component of the complex TFIIH), located in an area that presumably possesses the same structure of chromatin in all types of cells. The research was approved by the institute ethics governing board.

## Abbreviations

ACH: Active chromatin hub; bp: base pair; CEF: Chicken embryonic fibroblast; CTCF: CCCTC-binding factor; HS: Site of hypersensitivity to DNase I; LCR: Locus control region; RBC: Red blood cell; RT-qPCR: real-time reverse transcriptase polymerase chain reaction; 3C-qPCR: quantitative chromosome conformation capture assay.

## Competing interests

The authors declare that they have no competing interests.

## Authors’ contributions

SVU, AAG, and SVR designed the studies; SVU performed the studies; SVU, AAG, and SVR analyzed the data; and SVU, AAG, and SVR prepared the manuscript. All authors read and approved the final manuscript.
